# ﻿The Identity of *Rubuspekinensis* Focke and *R.crataegifolius* Bunge (Rosaceae)

**DOI:** 10.3897/phytokeys.195.83181

**Published:** 2022-04-28

**Authors:** Tiran Huang, Cong Wang, Juntao Li, Xuhang Liu, Lanqing Ma, Hong Wang

**Affiliations:** 1 College of Life Sciences, University of Chinese Academy of Sciences, Beijing 100049, China; 2 Beijing Advanced Innovation Center for Tree Breeding by Molecular Design, Beijing University of Agriculture, Beijing 102206, China; 3 College of Landscape Architecture, Beijing University of Agriculture, Beijing 102206, China

**Keywords:** Identity, new synonym, *R.crataegifolius*, Rosaceae, *Rubuspekinensis*

## Abstract

A critical examination of specimens, with literature and a field survey have shown that *Rubuspekinensis* is conspecific with *R.crataegifolius*. Their morphological variations range can be defined as: leaves at the base may be ovate, suborbicular, narrowly ovate, entire, at the middle, ovate, narrowly ovate, oblong-lanceolate, palmately 3-lobed or 5-lobed and at the top, ovate, lanceolate, entire or 3-lobed; flowers solitary in the axillae or several flowers clustered at the terminal of branchlets or formed into short racemes. Therefore, we treat the former species as a synonym for the latter one.

## ﻿Introduction

**Figure 1.**
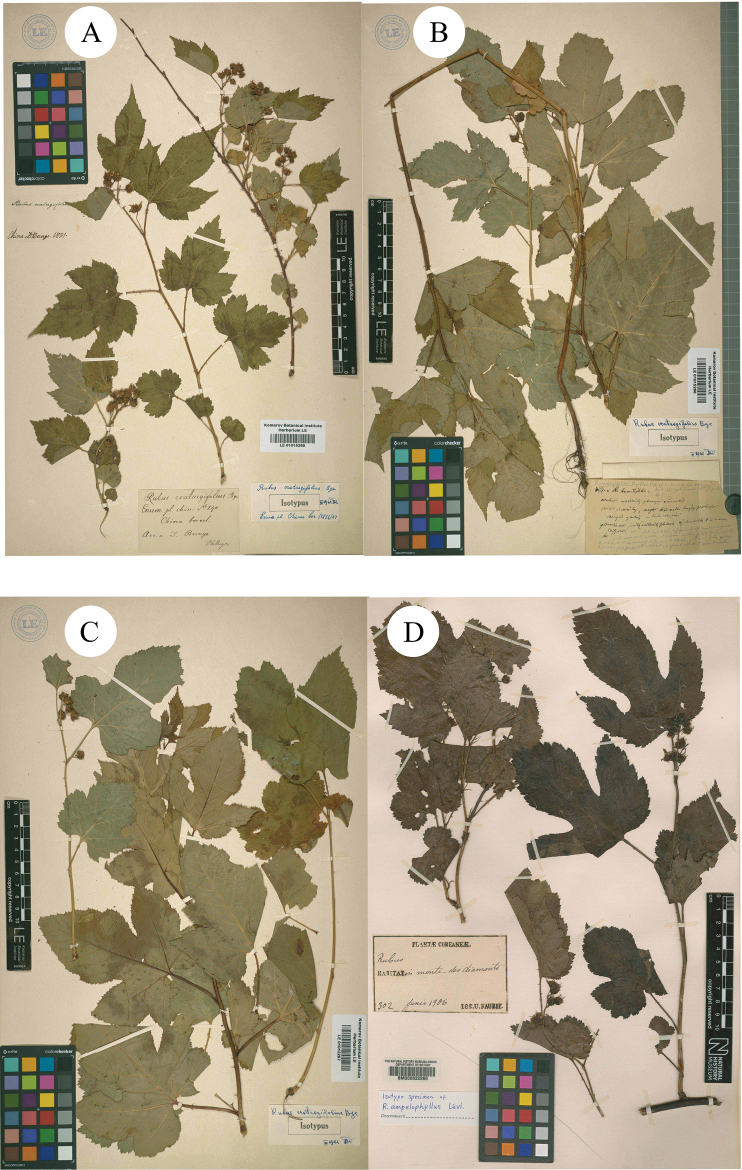
**A–C** syntypes of *R.crataegifolius***D** syntype of *R.ampelophyllus*.

*Rubuscrataegifolius*Bunge (1835: 98) was published, based on the collection from Pan-schan (Panshan), Hebei (now Tianjin), China A. Bunge s. n. (Syntypes LE [photo!], LE01015265, LE01015266, LE01015267, Fig. 1A–C). In the protologue, critical characteristics of the species were described as “Fruticosus erectus glabriusculus, ramis petiolis foliorum nervis pedicellisque aculeatis, foliis cordatis trifidis; lobis lateralibus acutis terminali acuminato acute inciso-dentatis, floribus axillaribus solitariis terminalibusque subracemosis; sepalis glabriusculis ovatis acuminatis erctis, petalis longe unguniculatis subspathulatis emarginatis, carpellis subexsuccis glabris”.

**Figure 2.**
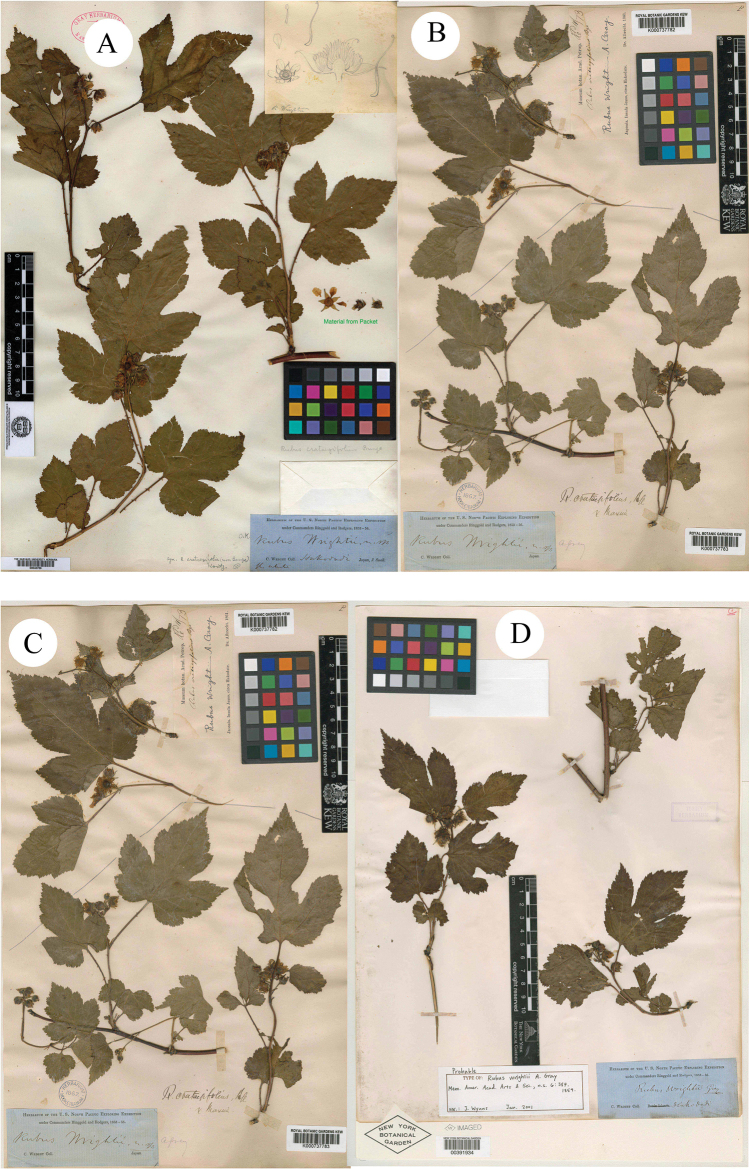
**A–D** syntypes of *R.wrightii*.

**Figure 3.**
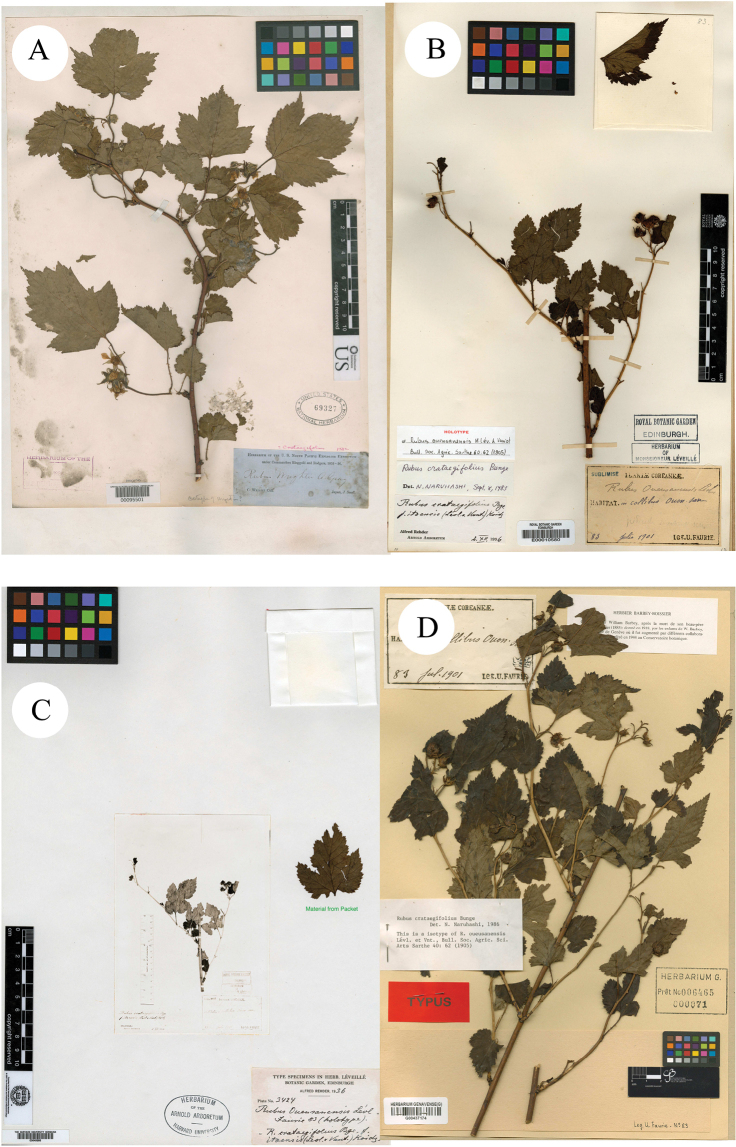
**A** syntype of *R.wrightii***B** holotype of *R.ouensanensis***C, D** isotypes of *R.ouensanensis*.

**Figure 4.**
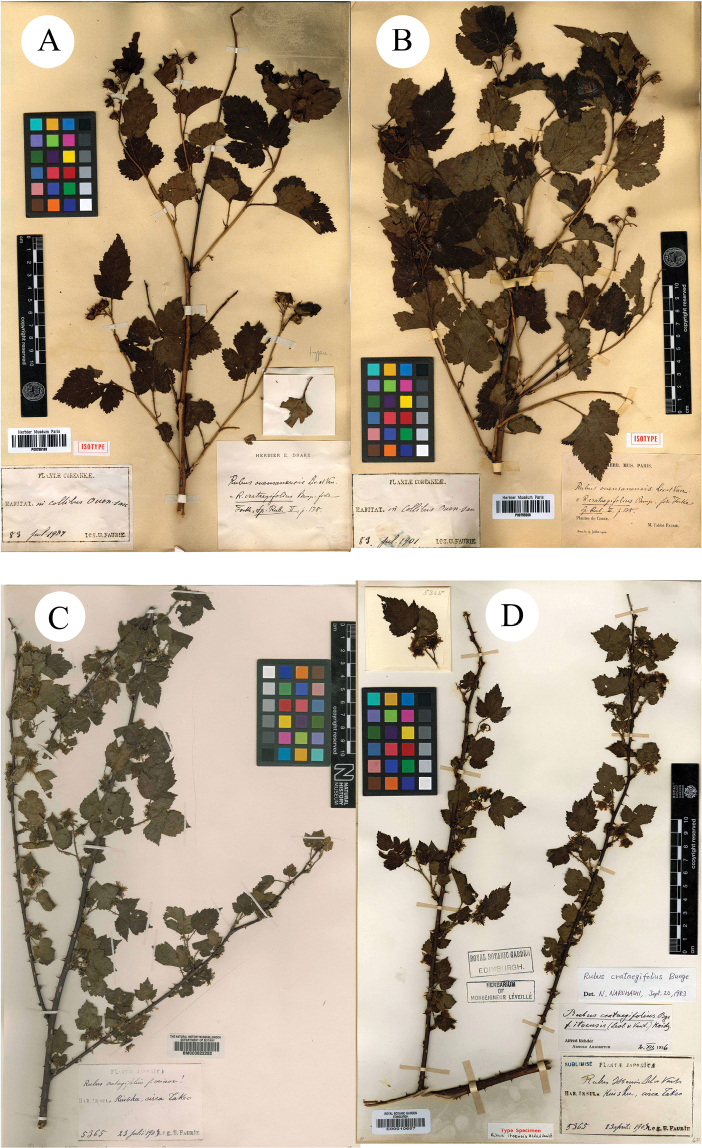
**A, B** isotypes of *R.ouensanensis***C, D** syntypes of *R.itoensis*.

A. Gray (1858: 387) described a new species *R.wrightii* A. Gray on the basis of specimens collected from Hokkaido, Japan (Syntypes GH [photo!] GH00040766; K [photo!] K000737782, K000737783; NY [photo!] NY00391934; US [photo!] US00095501, Figs 2A–D, 3A). H. Léveillé and Vaniot (1905: 62) described *R.ouensanensis* H. Lév. & Vaniot in Bull. Soc. Agric. Sarthe, based on the collection from Ouen san, Corea U. J. Faurie 83 (Holotype E [photo!] E00010580; Isotypes A [photo!] A00040688 (Image of E00010580); G [photo!] G00437174; P [photo!] P00755199, P00755200, Figs 3B–D, 4A, B). H. Léveillé (1908: 279) described *R.ampelophyllus* H. Lév. in Repert. Spec. Nov. Regni Veg., based on the collection from Quelpaert, Corea U. J. Faurie 302 (Syntype BM [photo!] BM000622269, Fig. 1D). These three species were treated as synonyms of *R.crataegifolius* by Lu (1985: 117) and Lu & Boufford (2003: 236) in Flora of China.

H. Léveillé and Vaniot (1905: 62) published a new species *R.itoensis* H. Lév. & Vaniot, based on the specimens collected from Takeo, Kiushu, Japan U. J. Faurie 5365 (Syntypes BM [photo!] BM000622252; E [photo!] E00010657, Fig. 4C, D). According to its protologue, this species was described as similar to *R.grossularia* H.Lév. & Vaniot and *R.incisus* Thunb., but it differed by having peduncles, with large and numerous flowers. However, it was treated as a synonym of *R.crataegifolius* in Flora of Japan (Naruhashi 2001).

**Figure 5.**
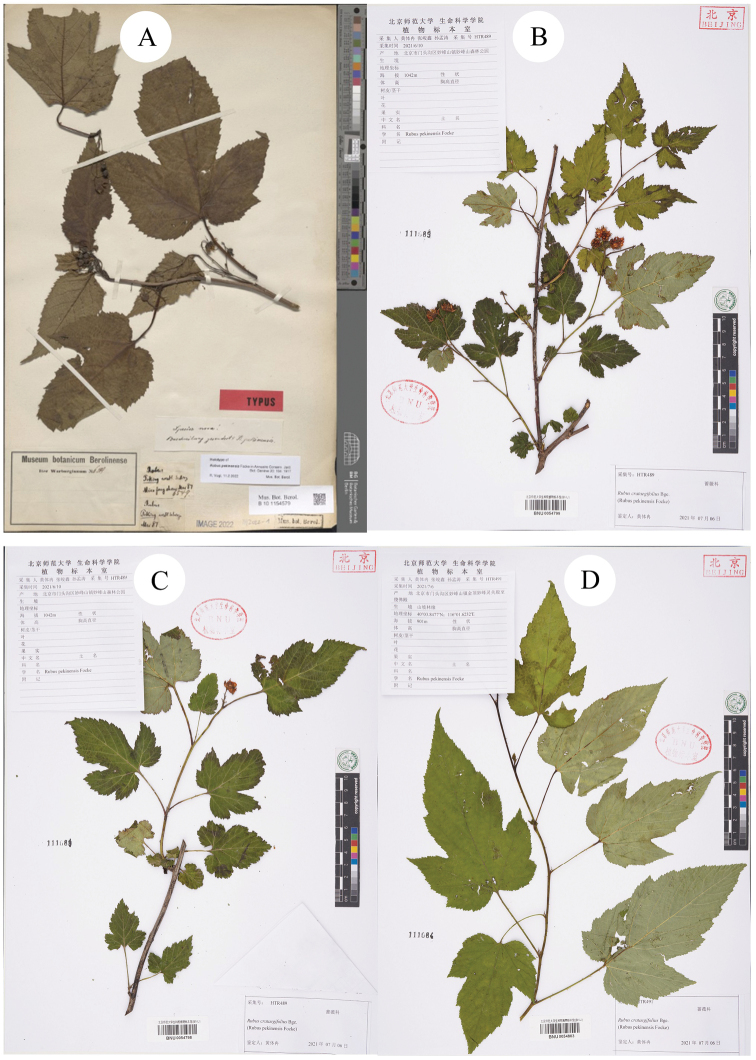
**A** holotype of *R.pekinensis***B–D** specimens of *R.pekinensis* collected from Miaofengshan.

Focke (1917: 104) described a new species *R.pekinensis* Focke on the basis of the collection of O. Warburg 6549 from Miaofangshan (Miaofengshan), Beijing, China (Holotype B [photo!] B101154579, Fig. 5A). According to the holotype, features of the specimen can be described as “shrubs with slightly curved prickles; leaves simple, 3-lobed or undivided, margin irregularly incised-serrate; stipules linear, adnate; several flowers cluster in axillae”. In the protologue, the characters of this species can be defined as: “Praesto est ramus exsiccatus unicus florens non incolumis speciem vero memorabilem indicans. Ramus foliorum marginibus a petiolis decurrentibus subangulatus, cum petiolis glabriusculus et aculeis lanceolatis vel falcatis sparsis armatus. Folia longe petiolata, majuscula, circ. 16 cm. longa, 18 cm. inter angulorum lateralium apices lata, inaequaliter sat grosse serrata, utrinque parce pilosa; folia inferiora subquinqueloba, intermedia e fundo cordato-emarginata triloba, inaequalia, lobis oblongo-lanceolatis; suprema integra, lanceolata. Stipulae petiolorum basi insertae, lineares. – Flores in ramulis axillaribus complures, longe pedunculati, alii superiores terminales fasciculati; pedunculi 3–6 cm. longi, laxe pilosi, interdum aculeolo instructi; calyces parce pilosi, sepalis saepe mucronatis vel appendice subulata terminatis; petala, ut videtur, sepala vix superantia.”. However, the name *R.pekinensis* has never been used, except in its original publication, from the date of its publication. Thus, it is necessary to identify the species.

## ﻿Materials and methods

Herbarium specimens, including all kinds of type specimens deposited in A, B, BM, E, G, GH, K, LE, NY, P and US were critically examined and a field survey in the type localities was conducted. Six populations from Miaofangshan and Pan-schan were surveyed and collected in 2021. High resolution pictures of leaves and fruits were taken by a SONY α 7II.

## ﻿Results

Firstly, the examination of herbarium specimens, identified as *R.ampelophyllus*, *R.crataegifolius*, *R.itoensis*, *R.ouensanensis* and *R.wrightii*, indicated that they represented the same species. According to Art. 11.4 of the “International Code of Nomenclature for algae, fungi, and plants (Shenzhen Code)” (Turland et al. 2018), *R.crataegifolius* is the correct name of this species. A comparison of type materials of *R.crataegifolius* and *R.pekinensis* showed that there are only a few differences in leaf morphology and inflorescence. So they also probably represented the same species.

**Figure 6.**
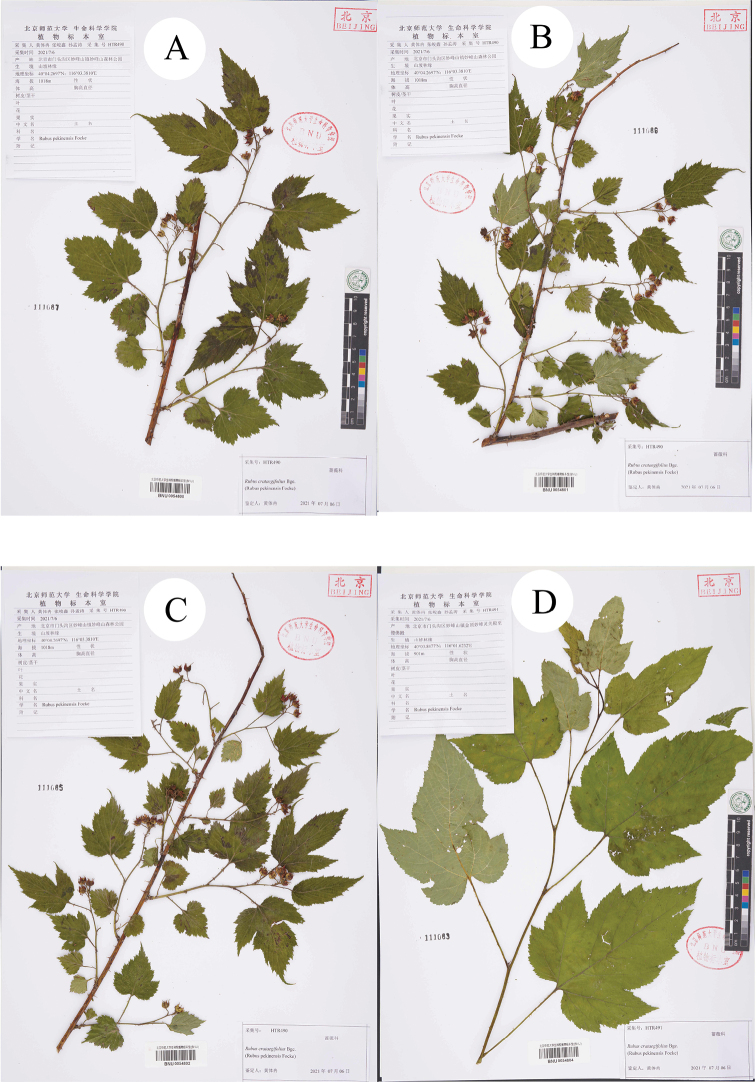
Specimens of *R.pekinensis* collected from Miaofengshan.

**Figure 7.**
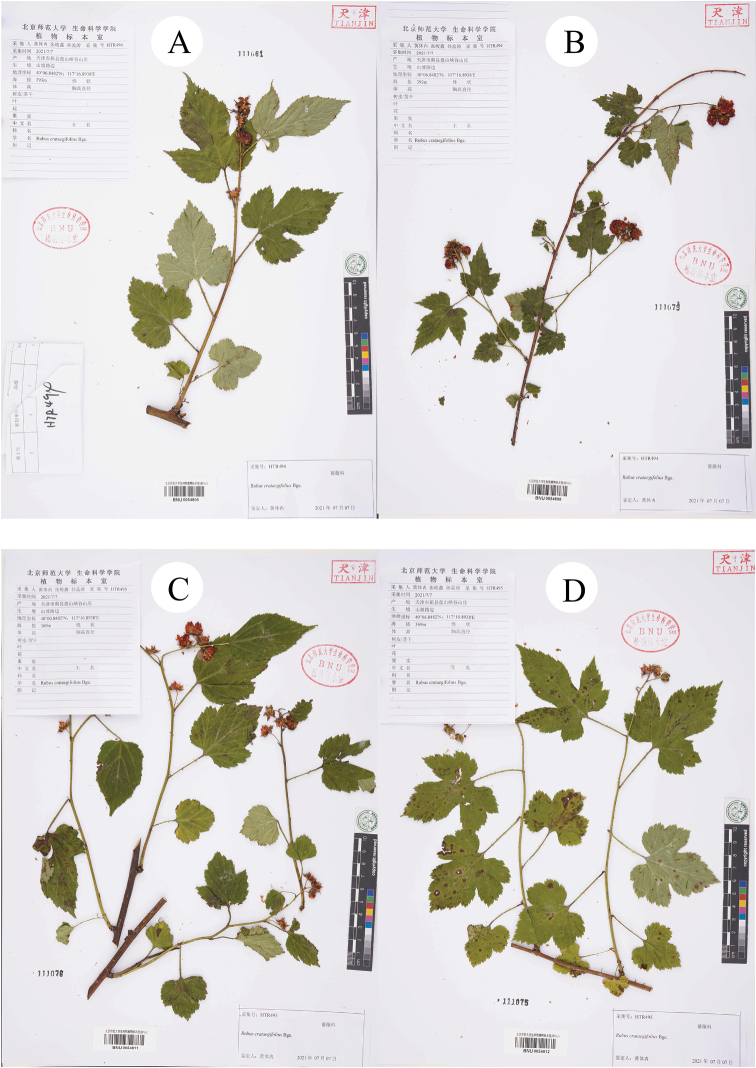
Specimens of *R.crataegifolius* collected from Panshan.

**Figure 8.**
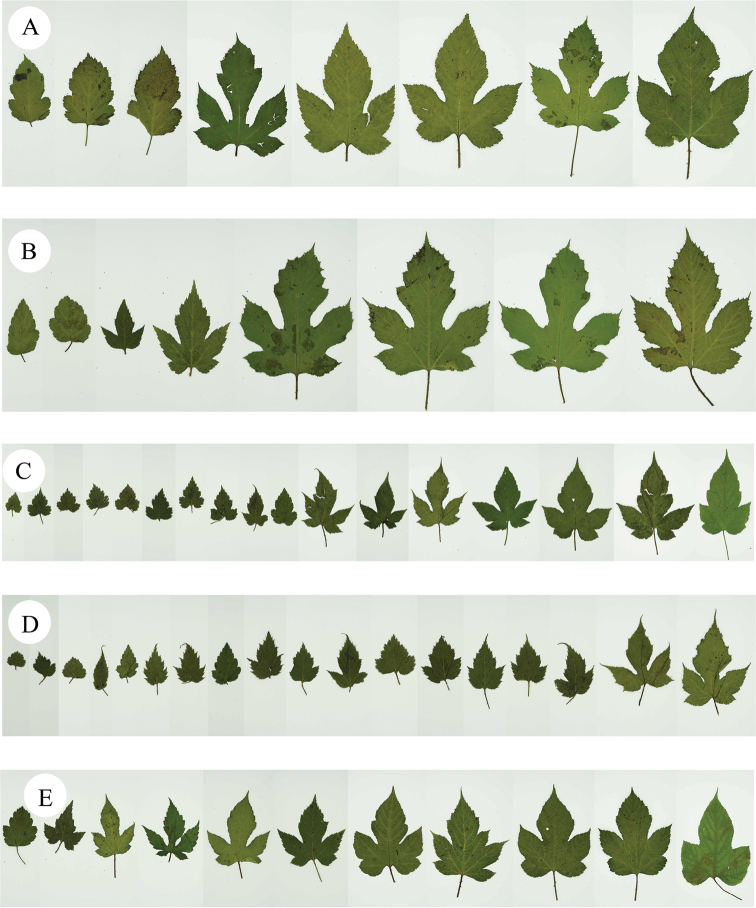
Morphological diversity of leaves **A, B** leaves of *R.crataegifolius*, collected from Panshan **C–E** leaves of *R.pekinensis*, collected from Miaofengshan.

Secondly, during the field survey to the type localities, three populations of the species *R.pekinensis* and *R.crataegifolius* were found from each location. All of the populations were at the fruiting stage and several observations regarding species identity were carried out in this *Rubus* group. The results can be described as: a) the morphological diversity of leaves is rich in different populations (Figs 1–6), even on the same plant (Fig. 8A–E). As Fig. 7 shows, leaves at the base may be ovate, suborbicular, narrowly ovate, entire, at the middle, ovate, narrowly ovate, oblong-lanceolate, palmately 3-lobed or 5-lobed and at the top, ovate, lanceolate, entire or 3-lobed. b) the diversity of inflorescence can be seen in *R.crataegifolius* and its synonyms, including *R.pekinensis*, such as the axillary flowers, solitary or several flowers clustered at the terminal of branchlets or formed into short racemes (Figs 1–6). c) the field survey in type localities of *R.pekinensis* and *R.crataegifolius* shows that they share characters of “branchlets angular, thinly pubescent when young, gradually glabrescent, with lanceolate or falciform prickles; leaves simple, middle-lower palmately 3–5-lobed, oblong-lanceolate, top entire lanceolate; stipules adnate to base of petiole, linear; flowers in branchlets axillary, others, terminal, short racemes or flowers several in a cluster; pedicel pubescent, sometimes equipped with aculeolus; calyx abaxially thinly pubescent, sepals narrowly ovate to ovate-oblong, apex acute to shortly acuminate, aggregate fruit red, glabrous” (Figs 5B–D, 6A–D, 7A–D). This is consistent with the protologue of Focke (1917: 104) and Bunge (1835: 98).

Based on the analysis above and the characters defined from the type specimen, we can confirm that *R.pekinensis*, together with *R.ampelophyllus*, *R.itoensis*, *R.ouensanensis* and *R.wrightii*, are conspecific with *R.crataegifolius*. As *R.pekinensis* was published later than *R.crataegifolius*, *R.pekinensis* should be a synonym of *R.crataegifolius*, according to nomenclatural priority.

## ﻿Taxonomic treatment

### ﻿ R. crataegifolius

Taxon classificationPlantaeRosalesRosaceae

Bunge Mém. Acad. Imp. Sci. St.-Pétersbourg Divers Savans 2: 98. 1835.

FB0390F3-402F-54CB-9A01-D3DC5146A891

R. crataegifolius Bunge Mém. Acad. Imp. Sci. St.-Pétersbourg Divers Savans 2: 98. 1835. Types: China, Hebei (now Tianjin): Pan-schan,1831, Bunge s. n. (Lectotype LE! LE 01015265 (designated here by Ti R. Huang); Isolectotypes LE!, LE01015266, LE01015267)
=R.pekinensis Focke Annuaire Conserv. Jard. Bot. Genève 20: 104.1917, syn. nov. Types: China, Beijing: Miaofangshan, May 1887, O. Warburg 6549 (Holotype B! B101154579).
=R.ampelophyllus H. Lév. Repert. Spec. Nov. Regni Veg. 5: 279. 1908. Types: Corea, Quelpaert, U. J. Faurie 302 (Holotype BM! BM000622269)
=R.itoensis H. Lév. & Vaniot Bull. Soc. Agric. Sarthe 60: 62. 1905. Types: Japan, Kiushu, circa Takeo, 13 April 1903, U.J. Faurie 5365 (Lectotype BM! BM 000622252 (designated here by Ti R. Huang); Isolectotype E! E 00010657)
=R.ouensanensis H. Lév. & Vaniot Bull. Soc. Agric. Sarthe 60: 62. 1905. Types: Corea, Ouen san, July 1901, U. J. Faurie 83 (Holotype E! E00010580; Isotypes A! A00040688 (Image of E00010580); G! G00437174; P! P00755199, P00755200)
=R.wrightii A. Gray Mem. Amer. Acad. Arts ser. 2, 6(2): 387. 1858. Types: Japan, Hokkaido, 1853, C. Wright s. n. (Lectotype GH! GH00040766 (designated here by Ti R. Huang); Isolectotypes K! K000737783; NY! NY00391934 US! US00095501)

#### Distribution and habitat.

*R.crataegifolius* grows on slopes, broad-leaved evergreen forests on hills, coniferous forests, thickets and roadsides. Its elevation ranges from 500–1000 m. In China, it is distributed in Anhui, Fujian, Guangxi, Jiangsu, Jiangxi and Zhejiang and overseas, in Japan.

#### Phenology.

Flowering from May to June and fruiting from July to August.

#### Taxonomic notes.

*R.crataegifolius* is similar to *R.chingii* H.H. Hu, the differences being: the latter leaves suborbicular, always palmately 5-parted, rarely 3- or 7-parted; flowers solitary; aggregate fruit densely hairy.

#### Additional specimens examined.

**China. Beijing.** 20 June 1964, Anren Li et al., no. 77 (PE00169287); June 1956, Herbarium, s. n. (TIE 00033553); 11 May 1930, T.N. Liou, no. 6874 (PE00169338); 30 June 1930, W.W. Smith, no. 1081 (PE00169344); **Heibei.** 1934, C.W.Wang, no. 61702 (IBSC0323698); 24 July 1984, Wuxiu Zhang et al., no. 0241 (PE01546856); 12 October 1957, Shaoying Qin, no. 38 (PE01466183); **Shandong.** 3 June 2013, Zhiyun Zhang & Lei Xie, 2013-032 (PE01979816); 1 May 2004, Yuantong Hou, no. 42011 (HITBC0008947); s.n. no.56047 (BNU002768); **Shanxi.** 29 May 1959, Kechien Kuan & Yilin Chen, no. 519 (PE00169384); 12 June 1959, Kechien Kuan & Yilin Chen, no. 684 (PE00169383); 6 July 1959, Kechien Kuan & Yilin Chen, no. 02128 (PE00169382 & PE00169385); 25 July 2014, Dongmei Kong, no. k0483 (PE02035182); 18 July 1986, Lanbin Guo, no. CLB0544 (BJFC00027073); **Tianjin.** 10 June 1984, Shiyuan He, no. 34234 (BNU 002746); 3 July 1956, Kechian Kuan, no. 1902 (PE 00169272); 9 July 1976, Jiayi Liu & Cailing Wang, no. JI0217 (TIE 00014583); 17 May 1985, Cailing Wang & Yongli Yu, no. JIN01446 (TIE 00014579); **Japan.** 3 August 1983, H. Migo, s.n. (NAS00368819, NAS00368820); 27 July 1928, Inagaki Kanichi, s.n. (NAS00368832); 1861, Albrecht, s.n. (K000737782); 1916, U.J. Faurie, 2370 (P03375389, P03375390, P03375391).

## ﻿Discussion

In the Flora of China, Lu and Boufford (2003: 284) indicated that *R.pekinensis* had been described from Peking (Beijing), China, but they have seen no specimens, and are therefore unable to treat it. Meanwhile, they stated that further revision of this species is necessary. In this paper, we carried out a field survey in the type locality from where morphological variations of *R.pekinensis* and *R.crataegifolius* in different populations were studied. There are many variations in the shapes of leaves both in *R.pekinensis* and *R.crataegifolius*, even in the same plant, where leaves simple, ovate, narrowly ovate, lanceolate or oblong-lanceolate, entire or palmately 3-lobed or 5-lobed, can be seen. Flowers solitary, clustered and short racemes sometimes co-existed in the same plant. This is consistent with the description of *R.crataegifolius* (Focke 1910, 1911, 1914). In further studies of *Rubus*, understanding the diversity of the species between different populations needs critical examination of specimens and field surveys, especially when a new species is being published.

## Supplementary Material

XML Treatment for R. crataegifolius
